# Proportion and reasons for loss to follow-up in a cohort study of people who inject drugs to measure HIV and HCV incidence in Kerman, Iran

**DOI:** 10.1186/s13011-021-00368-9

**Published:** 2021-04-01

**Authors:** Ghazal Mousavian, Nima Ghalekhani, Fatemeh Tavakoli, Willi McFarland, Armita Shahesmaeili, Heidar Sharafi, Mehrdad Khezri, Soheil Mehmandoost, Jasem Zarei, Hamid Sharifi, Ali Mirzazadeh

**Affiliations:** 1 HIV/STI Surveillance Research Center, and WHO Collaborating Center for HIV Surveillance, Institute for Futures Studies in Health, Kerman University of Medical Sciences, Kerman, Iran; 2 Department of Epidemiology and Biostatistics, University of California San Francisco, San Francisco, CA USA; 3 Middle East Liver Diseases (MELD) Center, Tehran, Iran

**Keywords:** Follow-up study, People who inject drugs, Loss to follow-up, Reminder system

## Abstract

**Background:**

Understanding the reasons for loss to follow-up (LTFU) in cohort studies, especially among marginalized groups such as people who inject drugs (PWID), is needed to strengthen the rigor of efficacy trials for prevention and treatment interventions. We assessed the proportion and reasons for loss to follow-up in a recent cohort of PWID enrolled in the southeast of Iran.

**Methods:**

Using respondent-driven sampling, we recruited 98 PWID age 18 years or older who reported injecting drugs in the past 6 months, and were negative for HIV and HCV at initial screening. Participants were followed at 6 week intervals, alternating a short six-week visit and long 12-week or quarterly visit to measure incidence of HIV and HCV. Methods to enhance retention included incentives for completing each visit, tracking people who missed the scheduled visits through their peer referral networks, engaged outreach teams to explore hotspots and residences, and photos. LTFU was defined as participants who missed their quarterly visits for two or more weeks.

**Results:**

Mean (SD) age of participants was 39.7 years (SD 9.6). Of 98 enrolled, 50 participants (51.0%) were LTFU by missed their scheduled quarterly visits for 2 weeks or more. For those whose reasons for LTFU could be defined (46.0%, 23 of 50), main reasons were: forgetting the date of visit (43.5%, 10 of 23), being incarcerated (39.1%, 9 of 23), and moving out of the city (17.4%, 4 of 23).

**Conclusion:**

This study highlighted the difficulty in retaining PWID in longitudinal studies. Despite having several retention strategies in place, over half of PWID were LTFU. The LTFU might be reduced by setting up more effective reminder systems, working closely with security systems, and online means to reach those who move outside the study area.

## Introduction

Injection drug use provides an efficient mechanism for transmitting blood borne viruses and in many low and middle-income countries like Iran, transmission among people who inject drugs (PWID) has emerged as a contributor to hepatitis C virus (HCV) and HIV epidemics through the sharing of drug injection equipment [1, 7, 15]. These two infections are responsible for high morbidity and mortality among PWID globally [6]. Approximately 15.6 million people inject drugs worldwide [9], with an estimated 208,000 residing in Iran [18]. Also the recent studies showed the prevalence of HIV and HCV among PWID (HIV: 14.3% before 2007 and 9.7% after 2007, HCV: 45%) [14, 20] and people who use non-injection drugs (HIV: 5.4% after 2005, HCV: 8%) remains considerably higher than those in the general population [2].

While studies have been primarily limited to cross-sectional studies among this key population in Iran where injection drug use is a significant route of HIV transmission, cohort studies of PWID are required to assess causal pathways from risk factors to acquisition of infection and, perhaps more importantly, to demonstrate the efficacy of interventions to prevent infection [4]. Randomized controlled trials for HIV and HCV prevention, including vaccine studies, need to be established. Direct measures of HIV and HCV incidence are only possible in prospective cohort studies [10]. Moreover, observational, community-based cohort studies of PWID for evaluating HIV and HCV vulnerabilities are lacking in many developing countries with high numbers of PWID, including Iran.

Loss to follow-up (LTFU) of participants is a main concern for internal validity of cohort studies, especially among hard-to-reach populations like PWID. LTFU not only decreases the power of the study (i.e., the sample size decreases), but also could lead to participation bias (those who remained are different from those who are lost or censored). Reasons for LTFU in cohorts of PWID in international studies [12, 23] included factors relating to transportation, such as money for fares and far distances to the study site [8, 24]. As the reasons for LTFU could be different in different settings, studies on the reasons for LTFU are necessary in different locations. To further improve our knowledge of barriers to retention of PWID for studies in Iran and possible extension to other areas of the Middle East, we characterize differences between PWID who were LTFU and those who remained in the early period of a cohort study in southeast of Iran.

## Methods

### Study design

This cohort study was conducted primarily to measure HIV and HCV incidence among PWID in Kerman city, located in the southeast of Iran. Participants were recruited from a parallel cross-sectional study that was used respondent-driven sampling (RDS) between July 10, 2018 and May 12, 2019. Eligibility criteria were: 1) injecting an illicit substance at least once in the last 6 months, 2) HIV and HCV antibody negative, 3) did not intend to travel from the city in the next 12 months, 4) being age 18 years or older, and 5) providing verbal consent. At baseline, participants were tested for HIV and HCV antibodies using rapid tests (SD BIOLINE HIV-1/2 3.0 and SD BIOLINE HCV). Reactive results for HIV and HCV were confirmed with fourth-generation enzyme-linked immunosorbent assay (ELISA) and reverse transcription- polymerase chain reaction (RT-PCR), respectively. Participants who confirmed to have HIV were referred to a voluntary counselling and testing (VCT) centre which facilitated their linkage to care. Participant who confirmed to have HCV were enrolled into a single-arm clinical trial for HCV care and treatment. HCV and HIV non-infected participants and those with sustained virologic response from the clinical trial arm were included in the cohort study to measure the incidence of these two infections.

### Data collection instrument

Demographic and risk behavioral data were collected using a face-to-face interview with two trained gender-matched interviewers in a private room. At the first visit, we used a bio-behavioral questionnaire which consisted of 14 sections, including socio-demographic characteristics, history of non-injection drug and alcohol use, smoking history, history of drug injection, history of addiction treatment, sexual risk behaviors, access and use of prevention programs, awareness of HIV transmission and prevention, HIV testing, awareness of HCV transmission and prevention, and mental health. A short questionnaire was used to update their behaviors in the past 3 months in follow-up interviews.

### Follow-up procedures and LTFU definition

Participants enrolled agreed to come to the study site at intervals of 6 weeks for almost 12 months. The first appointment was a short visit and with collection of 10 ml of blood for storage. The alternating quarterly appointments included a questionnaire measuring their behaviours during the last 12 weeks, in addition to collection of 10 ml of blood for storage and repeat rapid tests for HIV and HCV. Participants were received an additional $2.5 USD for completing short appointments and $6 USD for quarterly appointments. If participants missed their scheduled quarterly visits for 2 weeks or more, they were classified as LTFU. To mitigate LTFU, we engaged an outreach team familiar with hotspots and the registered addresses of the participants to find them and refer them to the study site. The outreach team, about 3 days before the scheduled visit, used a photo and phone number of the participants who had consented to use to assist with locating the participants. Further, we used the network of participants who were linked to recruitment to attempt re-contact (i.e., contact through their original recruiter or recruits). To measure reasons for LTFU, we included an additional interview using a structured questionnaire whenever people who missed the scheduled visits returned to the study site, or when reached through their networks or by the outreach team.

### Analysis

Data were collected using Research Electronic Data Capture (REDCap) software and were analyzed using Microsoft Excel 2013 and Stata 14.1 (StataCorp). Descriptive statistics characterized demographic and clinical information and tracing the history of participants who were LTFU. We used independent t-test and chi-square tests to examine associations between independent variables and LTFU status.

## Results

**Fig. 1:**
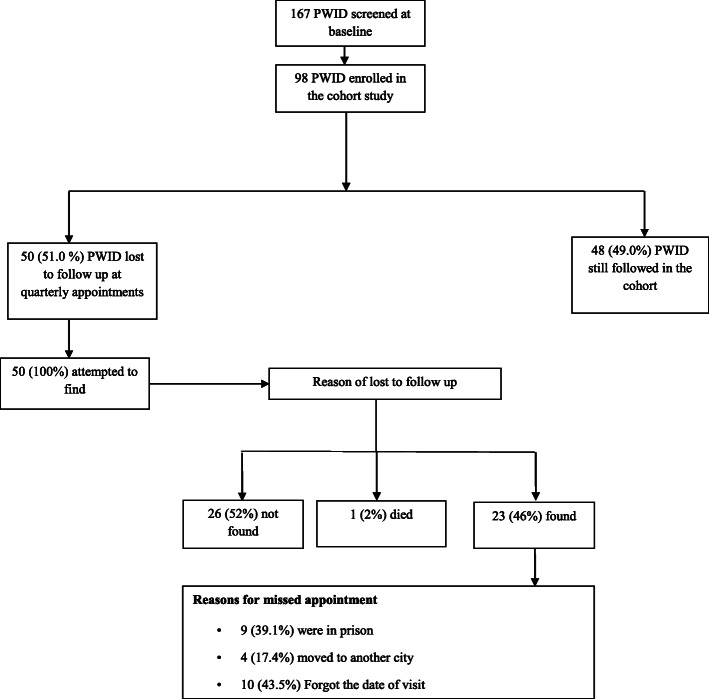
Reasons for LTFU in a cohort study among PWID in Kerman, Iran

A total of 167 eligible PWID were screened at the baseline visit. Of these, 98 agreed to participate in the cohort study (Fig. 1). The majority of participants were male (85.7%), 30 years of age or older (85.8%), ever experienced being homeless (91.8%), had some or completed secondary or high school education (543.1%), were not living with a spouse or partner (85.8%), employed (76.5%), and had a monthly income less than $100 (66.3%). While the majority had injected drugs with another person in the last 12 months (64.2%), sharing injecting equipment (8.1%) or syringes (6.1%) were low. Ever experiencing overdose was reported by (41.9%).

Of the 98 recruited PWID, 50 (51.0%; 95% confidence intervals (CIs): 38.1, 59.1) were LTFU. There was no significant difference between those who remained in the study and those who LTFU on any of the above variables apart from age (Table 1). Compared to those retained in the cohort, those LTFU were younger age (39.75 ± 9.64 vs. 37.96 ± 8.86 years, *p* = 0.049). Of the 50 LTFU we attempted to locate by all methods, we could not contact 26 persons (52.0%). One person (2%) had died. Among the 23 persons who we contacted, nine (39.1%) were incarcerated, four (17.4%) moved to another city, and ten (43.5%) said that they forgot the appointment date (Fig. 1).

**Table 1:** Demographic and behavior characteristics of participants in a cohort study among people who inject drugs in Kerman, Iran

Characteristics	Total (***N*** = 98)	Lost to Follow-up (***N*** = 50)	Retained in the cohort (***N*** = 48)	***P***-Value
**Sex**				
Female	14 (14.3%)	6 (12.0%)	8 (16.7%)	
Male	84 (85.7%)	44 (88.0%)	40 (83.3%)	0.509
**Age**				
Mean ± SD	39.75 ± 9.64	37.96 ± 8.86	41.79 ± 10.14	0.049
**Ever homeless**				
No	8 (8.2%)	4 (8.0%)	4 (8.5%)	0.927
Yes	90 (91.8%)	46 (92.0%)	43 (91.5%)	
**Education**				
Primary or less	37 (37.8%)	18 (36.0%)	19 (39.6%)	0.619
Some or completed secondary or high school	53 (54.1%)	29 (58.0%)	24 (50.0%)	
University	8 (8.1%)	3 (6.0%)	5 (10.4%)	
**Living with their spouse/partner**				
Yes (i.e., married, live with partners)	14 (14.2%)	8 (16.0%)	6 (12.5%)	
No (i.e., single, married but live alone, widowed)	84 (85.8%)	42 (84.0%)	42 (87.5%)	0.621
**Job Status**				
Unemployed	23 (23.5%)	10 (20.0%)	13 (27.1%)	
Employed	75 (76.5%)	40 (80.0%)	35 (72.9%)	0.408
**Monthly income**				
Under 1,000,000 T (<$100)	65 (66.3%)	34 (68.0%)	31 (64.6%)	0.303
1,000,000-4,999,999 T ($100–499)	31 (31.6%)	14 (28.0%)	17 (35.4%)	
5,000,000 T or more (>$500)	2 (2.1%)	2 (4.0%)	0	
**Alcohol use in the last 12 months**				
No	77 (79.4%)	36 (73.5%)	40 (85.1%)	0.160
Yes	20 (20.6%)	13 (26.5%)	7 (14.9%)	
**Injecting frequency reported in the last 3 months**				
No recent injection	15 (15.3%)	11 (22.5%)	4 (8.3%)	0.160
< weekly	34 (34.7%)	16 (32.6%)	18 (37.5%)	
< not daily but at least weekly (i.e., weekly)	22 (22.5%)	8 (16.3%)	14 (29.2%)	
Daily	27 (27.5%)	14 (28.6%)	12 (25.0%)	
**Injected with another person in the last 12 months**				
No	35 (35.8%)	18 (36.0%)	17 (36.2%)	0.986
Yes	63 (64.2%)	32 (64.0%)	30 (63.8%)	
**Receptive injecting equipment sharing in the last 12 months**				
No	90 (91.9%)	44 (88.0%)	46 (95.8%)	0.157
Yes	8 (8.1%)	6 (12.0%)	2 (4.2%)	
**Receptive syringe sharing in the last 12 months**				
No	92 (93.9%)	46 (92.0%)	46 (95.8%)	0.429
Yes	6 (6.1%)	4 (8.0%)	2 (4.2%)	
**Ever history of drug overdose**				
No	57 (58.1%)	26 (52.0%)	31 (64.6%)	0.207
Yes	41 (41.9%)	24 (48.0%)	17 (35.4%)	

While there were some suggested differences, we did not find significant difference in demographic and behaviors of those who were LTFU and found later compared to those who we could not find (Table 2).

**Table 2:** Demographic and behavior characteristics of people who inject drugs who loss to follow-up from a cohort study in Kerman, Iran

Characteristics	Total LTFU (***N*** = 50)	Not found (***N*** = 27)	Later Found (***N*** = 23)	***P***-Value
**Sex**				
Female	6 (12.0%)	2 (7.4%)	4 (17.4%)	
Male	44 (88.0%)	25 (92.6%)	19 (82.6%)	0.279
**Age**				
Mean ± SD	37.96 ± 8.86	39.59 ± 9.43	36.04 ± 7.91	0.719
**Ever homeless**				
No	4 (8.0%)	1 (3.7%)	3 (13.1%)	0.225
Yes	46 (92.0%)	26 (96.3%)	20 (86.9%)	
**Education**				
Primary or less	18 (36.0%)	11 (40.7%)	7 (30.5%)	0.624
Some or completed high school	29 (58.0%)	14 (51.9%)	15 (65.2%)	
University	3 (6.0%)	2 (7.4%)	1 (4.3%)	
**Living with their spouse/partner**				
Yes (i.e.,married, live with partners)	8 (16.0%)	6 (22.2%)	2 (8.7%)	
No (i.e., single, married but live alone, widowed)	42 (84.0%)	21 (77.8%)	21 (91.3%)	0.193
**Job Status**				
Unemployed	10 (20.0%)	5 (18.5%)	5 (21.7%)	
Employed	40 (80.0%)	22 (81.5%)	18 (78.3%)	0.777
**Monthly income**				
Under 1,000,000 T (<$100)	34 (68.0%)	20 (74.1%)	14 (60.8%)	0.252
1,000,000-4,999,999 T ($100–499)	14 (28.0%)	7 (25.9%)	7 (30.4%)	
5,000,000 T or more (>$500)	2 (4.0%)	0	2 (8.8%)	
**Alcohol use in the last 12 months**				
No	36 (73.5%)	19 (70.4%)	17 (77.3%)	0.586
Yes	13 (26.5%)	8 (29. 6%)	5 (22.7%)	
**Injecting frequency reported in the last 3 months**				
No recent injection	11 (22.5%)	7 (25.9%)	4 (18.2%)	0.291
< weekly	16 (32.6%)	9 (33.4%)	7 (31.8%)	
< not daily but at least weekly (i.e., weekly)	8 (16.3%)	6 (22.2%)	2 (9.1%)	
Daily	14 (28.6%)	5 (18.5%)	9 (40.9%)	
**Injected with another person in the last 12 months**				
No	18 (36.0%)	7 (25.9%)	11 (47.8%)	0.108
Yes	32 (64.0%)	20 (74.1%)	12 (52.2%)	
**Receptive injecting equipment sharing in the last 12 months**				
No	44 (88.0%)	24 (88.9%)	20 (87.0%)	0.834
Yes	6 (12.0%)	3 (11.1%)	3 (13.0%)	
**Receptive syringe sharing in the last 12 months**				
No	46 (92.0%)	26 (96.3%)	20 (87.0%)	0.225
Yes	4 (8.0%)	1 (3.7%)	3 (13.0%)	
**Ever history of drug overdose**				
No	26 (52.0%)	14 (51.8%)	12 (52.2%)	0.982
Yes	24 (48.0%)	13 (48.2%)	11 (47.8%)	

## Discussion

This study highlighted the difficulty of retaining PWID in a longitudinal study in Iran. Despite having several retention strategies, more than half of PWID missed their quarterly follow-up visit. The level of loss to follow-up in our study was higher than other studies. For example, one study of HIV incidence and factors contributing to retention in a 12-month follow-up study of PWID in Sichuan Province, China reported a retention rate of 70% [22]. Another study on HIV incidence and behavioral correlates of HIV acquisition among PWID in St Petersburg, Russia reported a retention rate of 80% [13].

Forgetting the date of visit, moved to another city, and incarceration, lack of transportation, distance, transfer to other similar studies with different objectives, financial constraints, and improving or deteriorating health were common reasons for not returning to study sites elsewhere [5, 8, 16]. Also, in the study of Weigel et al., out of 11,827 Patients who were lost to follow-up, 9432 (79.7%) had transferred to another clinic [25]. Our incentives attempted to pay a reasonable financial incentive to compensate for the expenses of participation and transportation costs. To reduce the LTFU, we tried to establish the study site in an easy-access place for the participants and the time for visiting was wide. Participants could attend the follow-up visits for 2 weeks (each week two possible visits) after their scheduled visit. Moreover, before running the study, we conducted a qualitative study to measure how we could reduce LTFU. Understanding the reasons for LTFU could help manage to reduce LTFU [17]. One challenge noted in the formative and follow-up phases was the likelihood of being detained and incarcerated. Among our study participants who missed their visits, over 40% were in prison. Alternatives to prison, such as sending PWID to drug abstinence camps, are being considered to decrease harm related to drugs [17]. Such solutions may also be more amenable to study participation follow-up.

Similar to other communities and countries, illicit drug use and users experience stigma and discrimination, having negative impact drug user’s mental and physical health. However, the stigmatization is not equal; studies showed that the use of a given substance is more stigmatized if it is injected rather than being inhaled or smoked [19, 21].

Similar to findings of other studies, we found that PWID may have experienced stigma and discrimination for injecting drugs by others or even among themselves, which decrease their willingness to seek for services or attending a study targeted for people who inject drugs that requires multiple visits. Any kind of link to such targeted services or studies may put them at risk of unwanted disclosure of their injecting drug status. They were also afraid that they would be arrested by police or that their families would find out that they inject drugs. In line with Arndt who mentioned that “There is no good reason to continue to support science built on this unintentional stereotyping”, we believe that strong evidences are need for proving our finding and we will consider this for our future studies [3]. To address these barriers, we should reduce stigma through education and awareness programs, using peer workers in research among PWID [17].

Although more than half of the participants were LTFU, there were no significant differences between LTFU and retained participants apart from age. Addressing LTFU is essential in two aspects. First, it will reduce the study’s power when the number of people who are missed is high. Second, this leads to participation bias, when those retained in a study differ from those who missed are different (informative censoring). The results of this study suggest that the LTFU would not greatly alter the risk profile of the cohort. When the missing is non-informative, the risk of participation bias is not a considerable concern. When the characteristics of those who retained in the study are the same as those who LTFU, the average outcome in the LTFU participants is the same as the average outcome in the retained participants. In these situations, we could estimate the parameters in LTFU participants and then in the whole population using some methods like inverse probability weighting [11].

This study has several limitations. First, the study’s sample size was small; therefore, the analysis may not have had enough power to detect smaller differences between those who retained and those who were LTFU. Second, behavioral data and the reasons for LTFU were self-reported. Some participants may not disclose their behaviors or reasons for LTFU due to participation bias and social acceptability bias. Third, we could not find the reasons for LTFU among more than half of the censored participants. Their reasons for lost to follow-up were therefore not known. Incorrect or missing telephone numbers and addresses were often the main reason why our team could not find them. Although our study had an outreach team that routinely tried to contact participants in hotspots, street locations, and other public spaces, the majority of participants who were LTFU could not be found.

## Conclusions

Despite limitations, this research is the first study in Iran to describe the reasons for LTFU of PWID in a cohort study. Also, it should be noted that the comprehensive formative assessment for evaluating the feasibility of conducting studies in a similar context like Iran is essential. We found the number of participants who missed their appointments was considerable, although differences between those LTFU and retained were not substantial. Improving reminder systems, working with prison organizations, recruiting people who have no definite plan to travel, and online means to contact those who do move are likely to improve retention in PWID population cohort studies.

## Data Availability

Data are available on request due to privacy or other restrictions.

## Abbreviations

LTFU: Loss to Follow Up
HIV: Human Immunodeficiency Virus
HCV: Hepatitis C Virus
SD: Standard Deviation
PWID: People Who Inject Drugs
RDS: Respondent-Driven Sampling
ELISA: Enzyme-Linked Immunosorbent Assay
RT-PCR: Transcription- Polymerase Chain Reaction
VCT: Voluntary Counselling and Testing
REDCap: Research Electronic Data Capture
NIMAD: National Institute for Medical Research Development
ITAPS: International Traineeships in AIDS Prevention Studies
